# Analysis of adverse drug reactions/events of cancer chemotherapy and the potential mechanism of Danggui Buxue decoction against bone marrow suppression induced by chemotherapy

**DOI:** 10.3389/fphar.2023.1227528

**Published:** 2023-08-15

**Authors:** Bin Yu, Xida Yan, Yuanying Zhu, Ting Luo, Muhammad Sohail, Hong Ning, Hui Xu

**Affiliations:** ^1^ NHC Key Laboratory of Nuclear Technology Medical Transformation, Department of Pharmacy, Mianyang Central Hospital, Mianyang, China; ^2^ Key Laboratory of Molecular Pharmacology and Drug Evaluation, School of Pharmacy, Collaborative Innovation Center of Advanced Drug Delivery System and Biotech Drugs in Universities of Shandong, Yantai University, Yantai, China; ^3^ School of Pharmacy, Southwest Medical University, Luzhou, China; ^4^ College of Pharmaceutical Sciences, Institute of Pharmaceutical, Zhejiang University, Hangzhou, China

**Keywords:** adverse reactions/events, bone marrow suppression, Danggui Buxue decoction, network pharmacology, molecular docking

## Abstract

**Objective:** To analyze the clinical characteristics of adverse reactions/events based on chemotherapy in cancer patients, and then explore the potential mechanism of Danggui Buxue Decoction (DBD) against chemotherapy-induced bone marrow suppression (BMS).

**Methods:** Retrospectively collected and evaluated were the clinical data of patients in a hospital who experienced adverse reactions/events brought on by chemotherapeutic medications between 2015 and 2022. We explored the potential mechanism of DBD against BMS using network pharmacology based on the findings of the adverse reactions/events analysis.

**Results:** 151 instances (72.25%) experienced adverse reactions/events from a single chemotherapy medication. Besides, platinum-based medications produced the most unfavorable effects. The study also found that chemotherapy caused the highest number of cases of BMS, including platinum drugs. Consequently, BMS is the most prevalent adverse reaction disease caused by chemotherapy found in this part. According to network pharmacology findings, DBD can prevent BMS primarily involving 1,510 primary targets and 19 key active ingredients. Based on the enrichment analysis, PI3K-AKT, TNF, MAPK, and IL-17 signaling pathways made up the majority of the DBD-resisting BMS pathways. Molecular docking displayed that kaempferol, the major active ingredient of DBD, had the highest binding energy (−10.08 kJ mol^-1^) with PTGS2 (a key target of BMS).

**Conclusion:** Cancer patients who received chemotherapy had a risk to develop BMS. Regular blood tests should be performed while taking medicine; early discovery and treatment can reduce a patient’s risk of experiencing adverse reactions/events. Additionally, this study demonstrated that DBD, through a variety of targets and pathways, may be crucial in avoiding BMS.

## 1 Introduction

In 2020, China would record 3 million new cancer deaths and 4.56 million new cancer patients, according to a report from the World Health Organization (Sung et al., 2021). Lung, colon, stomach, breast, and liver cancer had the highest incidence rates of all cancers. The top five causes of death were colon, lung, liver, gastric, and esophageal cancer. Furthermore, 3.21 million additional cancer deaths will occur in China in 2022, according to forecasts from the National Cancer Center of China and the Chinese National Cancer Clinical Research Center (Xia et al., 2022). Most tumors are still found most frequently in lung cancer. In contrast, the prevalence of esophageal, liver, and stomach cancer has steadily declined. However, the prevalence of bladder cancer, male prostate cancer, female breast cancer, and colon cancer has steadily climbed. The two main factors driving the rise in cancer fatalities are the population’s aging and the size of the adult population.

According to several studies, the primary methods used to treat tumors at the moment are surgical removal, pharmacological chemotherapy, radiotherapy, targeted therapy, immunotherapy, and traditional Chinese medicine (Bae et al., 2023; Eisenberg et al., 2023; Lazaroff and Bolotin, 2023; Song et al., 2023). Additionally, it is believed that medicinal chemotherapy is still the primary method of tumor treatment, in accordance with a range of different criteria and consensus both domestically and internationally (Chen et al., 2022; Reig et al., 2022; Strazzabosco et al., 2022). However, chemotherapy can kill and stop the growth of tumor cells, but it also has specific toxic and side effects on healthy human cells, which can easily result in some unavoidable adverse reactions/events like liver and kidney dysfunction, nausea, vomiting, headaches, chest tightness, and other reactions (Knezevic and Clarke, 2020; De Francia et al., 2022; Liu et al., 2022). Currently, there are numerous chemotherapy medications on the market today that not only harm the body but also interfere with their therapeutic effects, postponing or worsening the patient’s condition.

We used the data of cancer patients using chemotherapy drugs in a medical hospital from 2015 to 2022 as a sample to study and analyze the severity of adverse reactions/events to more clearly understand the occurrence, severity, and impact on patients of adverse reactions/events in chemotherapy drug antineoplastic treatment. Besides, Danggui Buxue Decoction (DBD), a traditional Chinese medicine, was also used in this study as an intervention method to investigate the potential mechanism of one adverse reaction/event disease brought on by chemotherapy drugs. The disease was based on the previous adverse reactions/events of cancer chemotherapy results, and it caused the most adverse reactions/events. This helped to further reveal the mechanism of DBD in preventing a certain injury disease caused by chemotherapy and provided theoretical support and a scientific foundation for in-depth clinical research and application (Sun et al., 2023; Zhao et al., 2023). Systems biology is the technological theory on which network pharmacology is built, which can be used to do network topology analysis, develop the drug target disease network relationship, discover medications for disease treatment, searche the network database, forecast the drug treatment mechanism, and create a thorough network study of medication effects from various levels and viewpoints starting with a multi-target research method (Hopkins AL., 2007; Li et al., 2022).

## 2 Materials and methods

### 2.1 Analysis of adverse drug reactions/events of chemotherapy

#### 2.1.1 Data collection

The clinical data of patients with adverse reactions/events in a hospital from 2015 to 2022 were retrospectively collected. The information on patients with adverse reactions/events was collected through the National Adverse Drug Reaction Monitoring System of China (2015–2017) and the Chinese Hospital Pharmacovigilance System (CHPS) (2018–2022).

#### 2.1.2 Inclusion and exclusion criteria

The criteria for inclusion and exclusion of patients were established as follows: Inclusion criteria: 1) All hospitalized tumor patients who have received a specific diagnosis of their tumor illness are included; 2) Chemotherapy medications are suspected to have been used by the included patients.

Exclusion criteria: 1) Non-cancer patients; 2) Suspected non-chemotherapeutic medication; 3) Radiation therapy was also administered at the time of admission. This criterion was used by two researchers to screen the entire procedure, and when there was a disagreement, a conclusion was taken after speaking with the third researcher.

#### 2.1.3 Content of information collected

The patient’s information was gathered, including 1) General patient data, such as age, gender, and underlying illnesses; 2) Suspected drug data, such as treatment regimen, combined medications, usage, and dosage; 3) Clinical manifestations of adverse reactions/events; and 4) The severity scale for adverse reactions/events.

#### 2.1.4 Evaluation criteria

According to the “Common Adverse Reaction Event Evaluation Standard (CTCAE), version 5.0” published by the US Department of Health and Human Services in 2017 the evaluation of adverse reactions/events was carried out (Freites-Martinez et al., 2021). Two researchers rated and categorized the degree of adverse reactions/events at the same time. In the event of a disagreement, it is settled by debate and bargaining with a third researcher. Grade 1, Grade 2, Grade 3, Grade 4, and Grade 5 were used to categorize the included patients’ adverse reactions/events according to the CTCAE criteria. Grades 1 and 2 are categorized as “general” whereas grades 3, 4, and 5 are categorized as “serious.” Additionally, according to the above criteria, other adverse reactions/events were classified.

#### 2.1.5 Statistical analysis

The data were statistically analyzed using SPSS 24.0 and Excel 2021, and the counting data were expressed in cases (*n*) and rates (%). Chi-square test was performed to compare the groups, and *p* < 0.05 denoted statistical significance. In addition, various information distributions (such as admission records, course records, test indicators, etc.) and other influencing factors were analyzed using the electronic case system of the hospital.

### 2.2 Network pharmacology and molecular docking

#### 2.2.1 DBD and disease target screening

Through the TCMSP platform to obtain the active ingredients of DBD, the keywords “Huangqi” and “Danggui” were searched, and the active ingredients of the compounds that were returned were screened. Moreover, suitable active ingredients were discovered under the screening parameters of OB% > 30% and DL > 0.18 (Wang et al., 2022). The Uniport protein database was utilized to standardize the targets following deduplication and screening.

The keyword “a certain injury disease caused by chemotherapy” was used to search the GeneCards and OMIM databases. Only targets with a score of 10 or higher are extracted from the first database, which is combined with the second database to retrieve related targets by eliminating duplicates. The potential key target is the point at which the associated target of “a certain injury disease caused by chemotherapy” and the target of the DBD active ingredient intersect.

#### 2.2.2 Construction of PPI and construction of an active ingredients-disease-target network

A protein-protein interaction (PPI) network is created by uploading the typical targets of DBD and BMS to the STRING platform and setting the species screening to “*Homo sapiens*” without completing combined score screening to study the interactions between the target proteins. Creating an active ingredient-disease-target network using Cytoscape and the pertinent target data from DBD and “a certain injury disease caused by chemotherapy.”

#### 2.2.3 GO function and KEGG pathway enrichment analysis

Through the DAVID database, GO analysis (three levels of biological processes, molecular functions, and cellular components) and pathways analysis were performed on the key targets of DBD for the treatment of “a certain injury disease caused by chemotherapy.” The results were saved and sorted out, and the top-ranked biological processes and signaling pathways were screened. Visual analysis was performed through Omicshare.

#### 2.2.4 Molecular docking

To verify the binding of active ingredients and key targets, AutoDock was utilized for the semi-flexible molecular docking of the ligand and receptor, and the AutoDock Vina scoring tool was used to calculate the free binding energy. The presence of a well-docked receptor and ligand was indicated when the free binding energy was less than −5.0 kJ mol^-1^ and the ability to bind is improved when the value is reduced (Niu et al., 2021). To illustrate the docking outcomes with minimal free binding energy, PyMOL software was employed.

#### 2.2.5 ADMET profiling

Chemical absorption, distribution, metabolism, excretion, and toxicity (ADMET) are particularly important and critical in drug discovery, development, and application. ADMET can assist in the efficient and safe analysis of the properties of discovered drugs. In this aspect, the physicochemical characteristics of the predicted ingredients were identified through two databases (SWISS ADME and admetSAR). They can be utilized to predict the toxicity of potential core ingredients of DBD, such as acute oral toxicity, aes mutation, carcinogenic, hepatotoxicity, and nephrotoxicity.

## 3 Results and analysis

### 3.1 Analysis of adverse reactions/events of chemotherapy drugs

#### 3.1.1 Screening of adverse reactions/events in cancer patients

**FIGURE 1:**
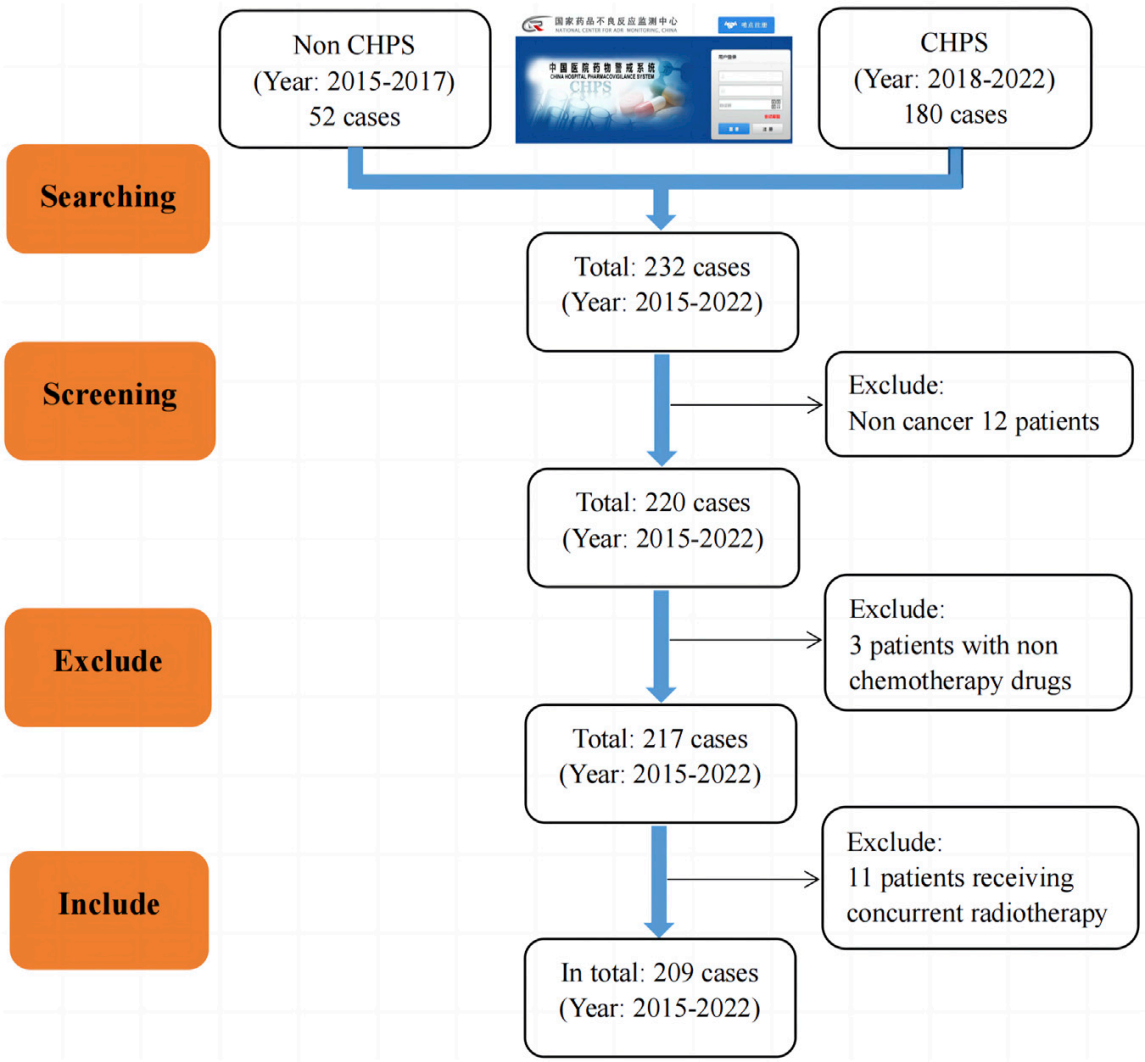
Flowchart for screening cases in two separate databases.

Between 2015 and 2022, the hospital reported a total of 232 patients through the National Adverse Drug Reaction Monitoring System and CHPS. According to the former, the incidence of adverse reactions/events decreased yearly from 2015 to 2017. However, since the CHPS system was launched in 2018, the number of reported adverse reactions/events has increased yearly except for 2020. In addition, out of a total of 232 cases included, 209 adverse reactions/events were ultimately included after screening according to the “1.1.2” criteria. The results are shown in Figure 1.

#### 3.1.2 Basic information and proportion of tumor patients included

In order to better understand the occurrence of adverse reactions/events after chemotherapy in male and female cancer patients at different ages, detailed statistics and analysis were conducted in this study, as shown in Table 1. Non-small cell lung cancer is the most prevalent tumor disease with the highest incidence of adverse reactions/events, accounting for 12.44%. The number of male patients are slightly more than female patients and they are older (over 60 years old). Age and gender among those with NSCLC differ statistically significantly (*p* < 0.001). The second, which accounts for 11.96%, is colon cancer. Patients who are female are slightly more numerous than patients who are male. Nevertheless, the majority of patients are under 60, and the age and gender differences are statistically significant (*p* < 0.001). Rectal cancer comes in third place and makes up more than 10% of cases. There are slightly more male patients and the age spans almost the entire age group, while female patients are mostly under the age of 60. Additionally, the ratio of male to female adverse reactions/events for non-Hodgkin’s lymphoma, acute non-lymphocytic leukemia, and gastric cancer is almost 1:1. Males were more likely to develop nasopharyngeal cancer, esophageal cancer, pancreatic cancer, Hodgkin’s lymphoma, and other adverse effects. Women’s attention was focused more on endometrial, breast, ovarian, and cervical cancer at the same time.

**TABLE 1:** Basic information and proportion of cancer patients.

Serial No.	Patients (*n*)	Gender (*n*)	<60 for age (%)	≥60 for age (%)	Tumor diseases	Proportion (%)	χ^2^ value	*p*-value
1	26	M: 15	4 (26.67)	11 (73.33)	Non-small cell lung cancer	12.44	26.000	<0.001
		F: 11	10 (90.91)	1 (9.09)				
2	25	M: 10	7 (70.0)	3 (30.0)	Colon cancer	11.96	25.000	<0.001
		F: 15	10 (66.67)	5 (33.33)				
3	22	M: 13	7 (53.85)	6 (46.15)	Rectal cancer	10.53	22.000	<0.001
		F: 9	7 (77.78)	2 (22.22)				
4	19	M: 11	7 (63.64)	4 (36.36)	Non Hodgkin’s lymphoma	9.09	19.000	<0.001
		F: 8	4 (50.0)	4 (50.0)				
5	15	M: 8	7 (87.50)	1 (12.50)	Acute non lymphocytic leukemia	7.18	15.000	<0.001
		F: 7	6 (85.71)	1 (14.29)				
6	12	M: 10	9 (90.0)	1 (10.0)	Nasopharyngeal carcinoma	5.74	12.000	<0.001
		F: 2	2 (100.0)	0 (0.0)				
7	11	M: 0	0 (0.0)	0 (0.0)	Cervical carcinoma	5.26	—	—
		F: 11	7 (63.64)	4 (36.36)				
8	11	M: 4	4 (100.0)	0 (0.0)	Acute lymphoblastic leukemia	5.26	11.000	<0.001
		F: 7	7 (100.0)	0 (0.0)				
9	8	M: 6	3 (50.0)	3 (50.0)	Esophageal cancer	3.83	8.000	0.005
		F: 2	2 (100.0)	0 (0)				
10	8	M: 0	0 (0.0)	0 (0.0)	Breast cancer	3.83	—	—
		F: 8	8 (100.0)	0 (0.0)				
11	6	M: 3	2 (66.67)	1 (33.33)	Gastric cancer	2.87	6.000	0.014
		F: 3	3 (100.0)	0 (0.0)				
12	6	M: 0	0 (0.0)	0 (0.0)	Oophoroma	2.87	—	—
		F: 6	5 (83.33)	1 (16.67)				
13	5	M: 4	3 (75.0)	1 (25.0)	Small cell lung cancer	2.39	5.000	0.025
		F: 1	0 (0.0)	1 (100.0)				
14	4	M: 4	2 (50.0)	2 (50.0)	Pancreatic cancer	1.91	—	—
		F: 0	0 (0.0)	0 (0.0)				
15	3	M: 1	1 (100.0)	0 (0.0)	Multiple myeloma	1.44	3.000	0.083
		F: 2	2 (100.0)	0 (0.0)				
16	3	M: 2	1 (50.0)	1 (50.0)	Bladder urothelial carcinoma	1.44	3.000	0.083
		F: 1	0 (0.0)	1 (100.0)				
17	2	M: 2	0 (0.0)	2 (100.0)	Hodgkin’s lymphoma	0.96	—	—
		F: 0	0 (0.0)	0 (0.0)				
18	2	M: 0	0 (0.0)	0 (0.0)	Epithelial carcinoma of the upper urinary tract	0.96	—	—
		F: 2	0 (0.0)	2 (100.0)				
19	2	M: 0	0 (0.0)	0 (0.0)	Renal cancer	0.96	—	—
		F: 2	0 (0.0)	2 (100.0)				
20	2	M: 0	0 (0.0)	0 (0.0)	Endometrial carcinoma	0.96	—	—
		F: 2	2 (100.0)	0 (0.0)				
21	17	M: 11	7 (63.64)	4 (36.36)	Others	8.13	19.000	<0.001
		F: 6	6 (100.0)	0 (0.0)				
Prop-ortion (%)	100.0							

M = male, F = female; “Others” includes cholangiocarcinoma, osteosarcoma, parotid gland cancer, chronic myelocytic leukemia, myelodysplastic syndrome, Fahrenheit macroglobulinemia, oropharyngeal cancer, chronic lymphocytic leukemia, glioma, urothelial carcinoma, prostate cancer, hypopharyngeal cancer, thymoma, penis cancer, primary unknown cervical lymph node metastatic squamous cell carcinoma, primary liver cancer, primitive neuroectodermal tumor, and 1 case each of the above tumor diseases.

#### 3.1.3 Combined medication

Among the 209 cases of adverse reactions/events caused by chemotherapy drugs included in this study, 151 cases were caused by a single use of drugs, 50 cases were caused by a combination of two drugs, 7 cases were caused by a combination of three drugs, and 1 case was caused by a combination of four drugs and above. In this part, single-drug use leads to the most significant proportion of adverse reactions/events in cancer patients. On the contrary, the combined use of drugs reduces the occurrence of adverse reactions/events to a certain extent, which plays an important role in the treatment effect and symptom relief of patients. As a result, when compared to single drug use, combination therapy results in fewer adverse reactions/events with chemotherapy drugs.

#### 3.1.4 Types of chemotherapy drugs

**FIGURE 2:**
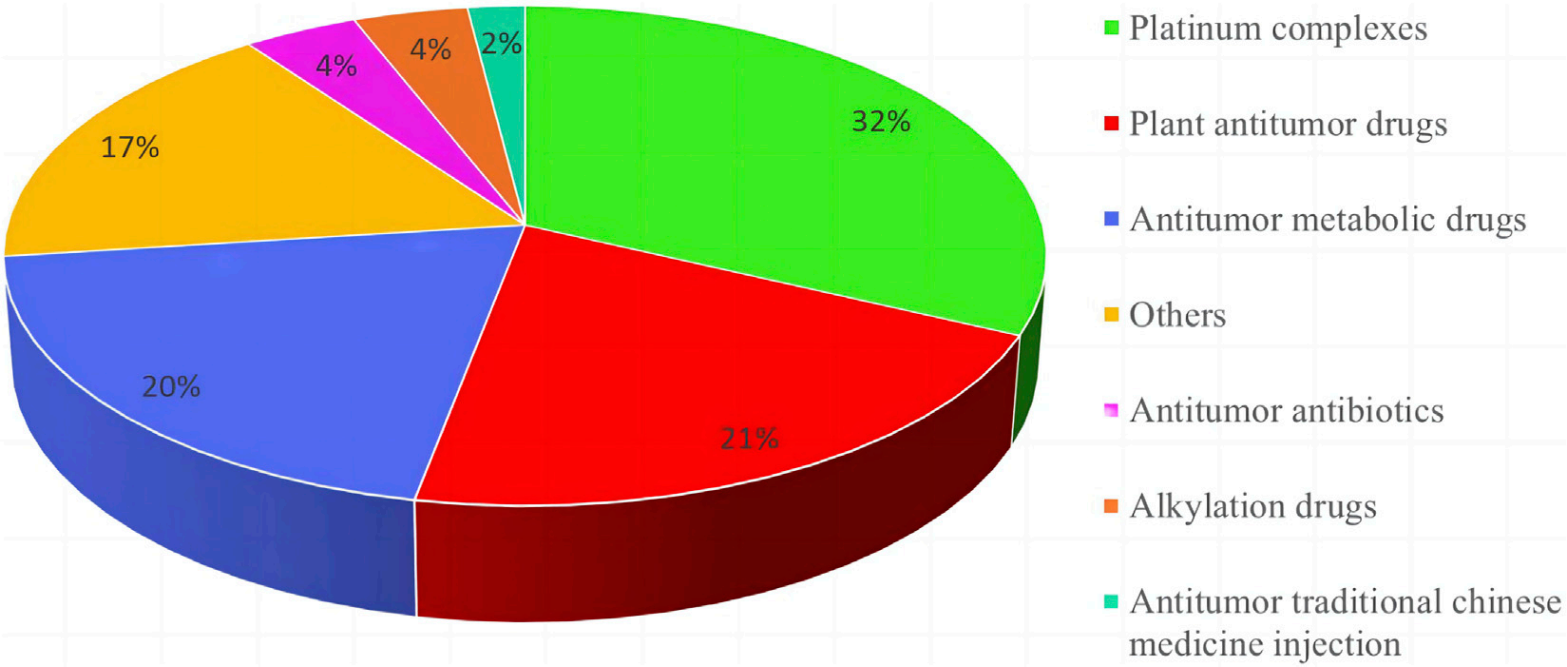
Classification and proportion of anti-cancer chemotherapy medications causing adverse reactions/events.

Among the 209 adverse reactions/events included in this study, nearly 30% of the patients had adverse reactions/events due to using 2 or more chemotherapy drugs, so the number of chemotherapy drugs was 271. To further understand the relationship between the types of chemotherapeutic drugs and adverse reactions/events to more clearly define the safety of clinical use of chemotherapeutic drugs, this study classified chemotherapeutic drugs according to the chemical structure and source of drugs. The results are shown in Figure 2. Among them, platinum complexes, plant anti-tumor drugs, and anti-tumor metabolic drugs caused the most adverse reactions/events in cancer patients, accounting for 32.0%, 20.0%, and 21.0%, respectively. At the same time, this study also counts the top 10 chemotherapy drugs that cause adverse reactions/events, with platinum complexes and paclitaxel accounting for the largest proportion. The detailed results are shown in Table 2.

**TABLE 2:** Top 10 chemotherapy drugs with the most adverse reactions/events.

Chemotherapy drugs	Cases(*n*)	Proportion (%)
Oxaliplatin	45	25.0
Cisplatin	36	20.0
Paclitaxel	28	15.56
Capecitabine	17	9.44
Cytarabine	17	9.44
Gemcitabine	12	6.67
Methotrexate	10	5.55
Tegio	5	2.78
Docetaxel	5	2.78
Etoposide	5	2.78
Total	180	100.0

#### 3.1.5 Severity of adverse reactions/events

The severity of clinical manifestations of adverse reactions/events in patients was rated based on CTCAE criteria. 76 “serious” adverse reactions/events and 279 “general” adverse reactions/events were recorded. Further investigation revealed that the “medical examination” category had the most cases, with 76 items, including 18 grade 1 and 25 grade 2 items in the “general” category, 27 grade 3 and 6 grade 4 items in the “serious” category, and no grade 5. Besides, the overall proportion of the “genera” category was relatively large, but most patients were tolerable and did not require special treatment.

There are 76 “serious” adverse reactions/events, of which “medical examination” has the highest frequency of clinical symptoms, with 33 items. In addition, there were 11, 9, and 7 “serious” adverse reactions/events related to “immune system diseases,” “respiratory, thoracic, and mediastinal diseases,” and “gastrointestinal diseases,” respectively. Therefore, for patients with “serious” adverse reactions/events, it is not only highly likely to be prolonged hospitalization events, limited activities of daily living, but also potentially disabling and life-threatening. Hence, special attention and care should be paid to “serious” adverse reactions/events. The detail can be seen in Table 3. Meanwhile, we observed that the frequency of BMS was higher in adverse reactions/events in Table 4, which were mostly characterized by a decline in neutrophil, white blood cell, and platelet counts. These findings also revealed that among the expected adverse reactions/events associated with chemotherapeutic medicines, BMS incidence and number were high.

**TABLE 3:** The severity of adverse reactions/events involving system organs of cancer patients.

Systemic organ classification	Number of clinical symptoms (*n*)	Severity [*n* (%)]			
		General		Serious	
		Grade 1	Grade 2	Grade 3	Grade 4
Medical examination	76	18 (23.68)	25 (32.89)	27 (35.53)	6 (7.90)
Respiratory, thoracic, and mediastinal diseases	56	11 (19.64)	36 (64.29)	9 (16.07)	0 (0.0)
Skin and subcutaneous tissue diseases	50	35 (70.0)	12 (24.0)	3 (6.0)	0 (0.0)
Gastrointestinal diseases	45	23 (51.11)	15 (33.33)	7 (15.56)	0 (0.0)
Nervous system diseases	26	7 (26.92)	16 (61.54)	3 (11.54)	0 (0.0)
The performance of general condition and medication site	24	17 (70.83)	5 (20.84)	2 (8.33)	0 (0.0)
Vascular disease	15	9 (60.0)	6 (40.0)	0 (0.0)	0 (0.0)
Skeletal muscle and connective tissue diseases	15	11 (73.33)	4 (26.67)	0 (0.0)	0 (0.0)
Heart disease	14	2 (14.29)	12 (85.71)	0 (0.0)	0 (0.0)
Immune system diseases	11	0 (0.0)	0 (0.0)	10 (90.91)	1 (9.09)
Kidney and urinary system diseases	7	1 (14.29)	2 (28.57)	4 (57.14)	0 (0.0)
Eye diseases	7	4 (57.14)	2 (28.57)	1 (14.29)	0 (0.0)
Blood and lymphatic diseases	5	2 (40.0)	1 (20.0)	2 (40.0)	0 (0.0)
Ear and labyrinthine diseases	3	0 (0.0)	2 (66.67)	1 (33.33)	0 (0.0)
Infection and infectious diseases	1	0 (0.0)	1 (100.0)	0 (0.0)	0 (0.0)
Total	355	140 (39.44)	139 (39.15)	69 (19.44)	7 (1.97)

**TABLE 4:** Proportion and clinical manifestations of severe adverse reactions/events in tumor patients.

Systemic organ classification	Number of clinical symptoms (*n*)	Grade 3 and grade 4(*n*)	Proportion (%)	Clinical manifestations (*n*)
Medical examination	76	33	43.42	Fever induced neutropenia (9), decreased white blood cell count (8), decreased neutrophil count (6), decreased platelet count (5), increased alanine aminotransferase (1), elevated serum creatinine (1), elevated serum bilirubin (1), neutropenia (1), and weight gain (1)
Respiratory, thoracic and mediastinal diseases	56	9	16.07	Dyspnea (5), laryngeal edema (1), abnormal pulse (1), Bronchospasm (1), bronchial stenosis (1)
Skin and subcutaneous tissue diseases	50	3	6.0	Hives (1), hyperhidrosis (1), suppurative dermatitis (1)
Gastrointestinal diseases	45	7	15.56	Intestinal obstruction (3), vomiting (2), constipation aggravation (1), diarrhea (1)
Nervous system diseases	26	3	11.54	Peripheral Sensory nerve disorders (2), syncope (1)
The performance of general condition and medication site	24	2	8.33	Fever (1), edema (1)
Immune system diseases	11	11	100.0	Allergic reaction (9), anaphylaxis (2)
Kidney and urinary system diseases	7	4	57.14	Acute renal failure (2), renal insufficiency (2)
Eye diseases	7	1	14.29	Eye congestion (1)
Blood and lymphatic diseases	5	2	40.0	Secondary anemia (1), anemia (1)
Ear and labyrinthine diseases	3	1	33.33	Vertigo (1)

#### 3.1.6 Chemotherapy drugs with serious adverse reactions/events

According to the results of “3.1.4” and “3.1.5,” a total of 76 serious adverse reactions/events were reported. To further clarify which chemotherapy drugs in this study have caused more serious adverse reactions/events, our team analyzed the reports of serious adverse reactions/events caused by the top 10 chemotherapy drugs, Among them, oxaliplatin and cisplatin, the platinum complexes, ranked in the top two places. The clinical manifestations of cancer patients are respectively concentrated in white blood cell count reduction (3 times), neutrophil count reduction (3 times), allergic reactions (2 times), dyspnea (2 times), as well as white blood cell count reduction (3 times), neutrophil count reduction (2 times), and renal insufficiency (2 times), etc. The 11 serious adverse reactions/events caused by paclitaxel were manifested in allergic reactions (5 times), dyspnea (3 times), and neutrophil count reduction (2 times). After paclitaxel, capecitabine was successively ranked, with 6 serious reports. There have also been five serious reports of etoposide. The detailed results can be seen in Table 5, and gemcitabine had no serious adverse reactions/events. In addition, we have also found an interesting phenomenon: in the statistical reports of serious adverse reactions/events caused by the top 10 chemotherapy drugs, almost all of the chemotherapy drugs have a reduction in white blood cells, neutrophils, or platelet counts. These results indicated that these chemotherapy drugs can cause adverse reactions/events in the cancer patient’s blood system, which will lead to the occurrence of BMS. The result was also consistent with Table 4, where the number of cases and incidence of BMS caused by chemotherapy drugs were the highest. DBD has a protective effect on the hematopoietic system of chemotherapy induced BMS, but the specific mechanism is not clear (Kong et al., 2022). Hence, we will utilize the current popular network pharmacology method to explore the potential mechanism of DBD against BMS in the next step.

**TABLE 5:** Top 10 varieties of chemotherapy drugs with severe adverse reactions/events.

Ranking	Drug variety	Number of level 3 severe reports (*n*)	Number of level 4 severe reports (*n*)	Clinical manifestations (*n*)
1	Oxaliplatin	15	2	Neutrophil count decrease (3), white blood cell count decrease (3), allergic reaction (2), dyspnea (2), laryngeal edema (1), suppurative dermatitis (1), syncope (1), ocular congestion (1), Hives (1), peripheral Sensory nerve disturbance (1), intestinal obstruction (1)
2	Cisplatin	10	1	Neutropenia (3), decreased white blood cell count (2), neutropenia with fever (1), renal insufficiency (2), acute renal failure (1), allergies (1), vomiting (1)
3	Paclitaxel	10	1	Allergic reactions (5), dyspnea (3), neutropenia (2)
4	Capecitabine	6	0	Intestinal obstruction (3), renal insufficiency (1), decreased neutrophil count (1), decreased platelet count (1)
5	Etoposide	5	0	Reduced neutrophil count (2), hyperhidrosis (1)、intestinal obstruction (1), allergic reactions (1)
6	Methotrexate	3	2	Acute renal insufficiency (1), anemia (1), edema (1), decreased platelet count (1), decreased neutrophil count (1)
7	Cytarabine	4	0	Neutropenia (2), pulse abnormalities (1), allergic reactions (1)
8	Docetaxel	2	1	Reduced white blood cell count (3)
9	Tegio	2	0	Reduced neutrophil count (1), diarrhea (1)

### 3.2 Network pharmacology

The second section was based on the BMS caused by chemotherapy, which was a significant and common adverse reaction or event disease, that was discovered in the previous study (“3.1” analysis of adverse reactions/events of chemotherapy drugs).

#### 3.2.1 DBD and disease target screening

Through the TCMSP database and conditional search of DBD, 22 effective components were obtained. Deduplication and exclusion of 3 unrelated target components resulted in a total of 19 active ingredients being included. Among them, Huangqi has 2 active ingredients, and Danggui has 17 active components. After weight removal, there are a total of 229 related targets. The results are shown in Table 6. 8670 BMS-related targets were found in the GeneCards database, but only 1,319 targets with a relevant score of 10 or above were included. Furthermore, 230 BMS targets were obtained from the OMIM database, and 1510 BMS-related targets were included in these two databases’ combination and de-duplication.

**TABLE 6:** Active ingredients information of DBD.

Molecule ID	Molecule name	Molecular structure	OB(%)	DL	Orginin
MOL000358	Beta-sitosterol	PGR, NCOA2, PTGS1, PTGS2, HSP90, PIK3CG, KCNH2, PPARG, DRD1, CHRM3, CHRM1, SCN5A, GABRA2, CHRM4, PDE3A, HTR2A, GABRA5, ADRA1A, GABRA3, CHRM2, ADRA1B, ADRB2, CHRNA2, SERT, OPRM1, GABRA1, CHRNA7, CAMC, BCL2, BAX, CASP9, NAPA, CASP3, CASP8, PRKCA, TGFB1, PON1, MAP2	36.91	0.75	Danggui
MOL000449	Stigmasterol	PGR, MCR, NCOA2, ADH1C, IGHG1, RXRA, NCOA1, PTGS1, PTGS2, ADRA2A, SLC6A2, SLC6A3, ADRB2, AKR1B1, PLAU, LTA4H, MAOB, MAOA, PRKACA, CTRB1, CHRM3, CHRM1, ADRB1, SCN5A, HTR2A, ADRA1A, GABRA3, CHRM2, ADRA1B, GABRA1, CHRNA7	43.83	0.76	Danggui
MOL000211	Mairin	PGR	55.38	0.78	Huangqi
MOL000239	Jaranol	NOS2, PTGS1, AR, SCN5A, PTGS2, ESR2, DPP4, HSP90AA1, CDK2, CHK1, TRY1, NCOA1, CALM	50.83	0.29	Huangqi
MOL000296	Hederagenin	PGR, NCOA2, CHRM3, CHRM1, GABRA2, GABRA3, CHRM2, ADRA1B, GABRA1, GRIA2, GABRA6, GABRA5, IGHG1, ADH1B, ADH1C, LYZ, COBT, PTGS1, SCN5A, PTGS2, RXRA, PDE3A, SLC6A2, CAMC	36.91	0.75	Huangqi
MOL000033	(3S,8S,9S,10R,13R,14S,17R)-10,13-dimethyl-17-[(2R,5S)-5-propan-2-yloctan-2-yl]-2,3,4,7,8,9,11,12,14,15,16,17-dodecahydro-1H-cyclopenta[a]phenanthren-3-ol	PGR	36.23	0.78	Huangqi
MOL000354	Isorhamnetin	NOS2, PTGS1, ESR1, AR, PPARG, PTGS2, PTP1B, ESR2, DPP4, MAPK14, GSK3B, HSP90AA1, CDK2, PIK3CG, PRKACA, TRYP1, PIM1, CCNA2, NCOA2, CALM, PYGM, PPARD, CHEK1, AKR1B1, NCOA1, F7, TR, NOS3, ACHE, GABRA1, MAOB, GRIA2, CALM, RELA, XDH, Ncf1, OLR1	49.60	0.31	Huangqi
MOL000371	3,9-di-O-methylnissolin	NOS2, PTGS1, CHRM3, TR, CHRM1, ESR1, ADRB1, SCN5A, PTGS2, NOS3, HTR3A, Adra2c, RXRA, ACHE, PDE3A, ADRA1B, ADRB2, ADRA1D, OPRM1, GABRA1, TRYP1, NCOA2, CALM	53.74	0.48	Huangqi
MOL000378	7-O-methylisomucronulatol	NOS2, PTGS1, DRD1, CHRM3, TR, KCNC2, CHRM1, ESR1, AR, ADRB1, SCN5A, PPARG, GAS6, CHRM5, PTGS2, NOS3, ADRA2C, CHRM4, RXRA, OPRD1, PDE3A, HTR2A, ADRA1A, CHRM2, ADRA1B, SLC6A3, ADRB2, ADRA1D, SERT, ESR2, GABRA1, DPP4, MAPK14, GSK3B, HSP90AB1, CDK2, CHEK1, PRKACA, RXRBB, TRYP1, PIM1, CCNA2, NCOA2, KCNMA1, CAM	74.69	0.30	Huangqi
MOL000379	9,10-dimethoxypterocarpan-3-O-β-D-glucoside	PTGS2, TOP2A, NCOA2	36.74	0.92	Huangqi
MOL000380	(6aR,11aR)-9,10-dimethoxy-6a,11a-dihydro-6H-benzofurano[3,2-c]chromen-3-ol	NOS2, PTGS1, CHRM3, TR, CHRM1, ESR1, SCN5A, PTGS2, HTR3A, RXRA, ACHE, ADRA1B, ADRB2, ADRA1D, GABRA1, HSP90AB1, CHRNA7, TRYP1, NOCA1, NCOA2, CAM, CHRM4	64.26	0.42	Huangqi
MOL000387	Bifendate	PTGS2, VEGFR2, FGFR, HSP90AB1, KCNMA1, PTGS1, TOP2	31.1	0.67	Huangqi
MOL000392	Formononetin	NOS2, PTGS1, CHRM1, ESR1, AR, PPARG, PTGS2, RXRA, PDE3A, ADRA1A, SLC6A3, ADRB2, SERT, ESR2, DPP4, MAPK14, GSK3B, HSP90AB1, CDK2, MAOB, CHEK1, PRKACA, TRYP1, PIM1, CCNA2, CAM, PKIA, TR, NOS3, ACHE, AMPC, JUNB, PPARG, IL4, SIRT1, ATP5F1B, MTND6, HSD3B, HSD3B1	69.67	0.21	Huangqi
MOL000417	Calycosin	NOS2, PTGS1, ESR1, AR, PPARG, PTGS2, RXRA, PDE3A, ESR2, DPP4, MAPK14, GSK3B, HSP90AB1, CDK2, CHEK1, PRKACA, TRYP1, PIM1, CCNA2, NCOA2, CAM, ADRB2	47.75	0.24	Huangqi
MOL000422	Kaempferol	NOS2, PTGS1, AR, PPARG, PTGS2, HSP90AB1, PIK3CG, PRKACA, NCOA2, DPP4, TRYP1, TR, CHRM1, NOS3, GABRA2, ACHE, SLC6A2, CHRM2, ADRA1B, GABRA1, TOP2, F7, CALM, RELA, IKBKB, AKT1, BCL2, BAX, TNF, NAPA, AHSA1, CASP3, MAPK8, XDH, MMP1, STAT1, CDC2, PPARG, HMOX1, CYP3A4, CYP1A2, CYP1A1, ICAM1, SELE, VCAM1, NR1I2, CYP1B1, ALOX5, HAS2, GSTP1, AHR, PSMD3, SLC2A2, NR1I3, INSR, DIO1, PPP3CB, PRXC1A, GSTM1, GSTM2, AKR1C3, SLPI	41.88	0.24	Huangqi
MOL000433	FA	CDK2, TR, GSK3B	68.96	0.71	Huangqi
MOL000439	Isomucronulatol-7,2'-di-O-glucosiole	TOP2A	49.28	0.62	Huangqi
MOL000442	1,7-Dihydroxy-3,9-dimethoxy pterocarpene	PTGS2, RXRA, HSP90AB1, TRY1	39.05	0.48	Huangqi
MOL000098	Quercetin	PTGS1, AR, PPARG, PTGS2, HSP90AB1, PIK3CG, NCOA2, DPP4, AR, TRY1, TOP2, TR, KCNH2, SCN5A, GAS6, ADRB2, MMP3, PRKACA, F7, NOS3, RXRA, ACHE, GABRA1, MAOB, RELA, AKT1, VEGFA, CCND1, BCL2, BCL2L1, FOS, CDK1, EIF6, BAX, CASP9, PLAU, MMP2, MMP9, MPK1, IL10, EGF, RBL1, TNF, NAPA, IL6, CDKN2A, AHSA1, CASP3, TP53, ELK1, NFKBIA, POR, ODC1, XDH, CASP8, TOP1, RAF1, SOD, PRKCA, MMP1, HIF1A, STAT1, RUNX1T1, LOC103184504, CDC2, HELS89N, ERBB2, PPARG, ACC1, HMOX1, CYP3A4, CYP1A2, CAV1, MYC, F3, GJA1, CYP1A1, ICAM2, IL1B, CCL2, SELE, VCAM1, PTGER3, IL8, PRKCB, BIRC5, DUOX2, NOS3, HSPB1, TGFB1, SULT1E1, MGAM, IL2, NR1I2, CYP1B1, CCNB1, PLAT, THBD, SERPINE1, COL1A1, IFNG, ALOX5, PTEN, IL1A, MPO, TOP2A, NCF1, ABCG2, HAS2, GSTP1, NFE2L2, NQO1, PARP1, AHR, PSMD3, SLC2A4, COL3A1, GYRB, CXCL11, CXCL2, DCAF5, NR1I3, CHEK2, INSR, CLDN4, PPARA, PPARD, HSPB1, CRP, CXCL10, CHUK, SPP1, RUNX2, RASSF1, E2F1, E2F2, ACP3, CTSD, IGFBP3, IGF2, CD40LG, IRF1, ERBB3, PON1, DIO1, PCOLCE, NPEPPS, HK2, NKX31, RASA1, PRXC1A, GSTM2, GSTM1	46.43	0.28	Huangqi

#### 3.2.2 Construction of PPI and active ingredient-disease-target network

**FIGURE 3:**
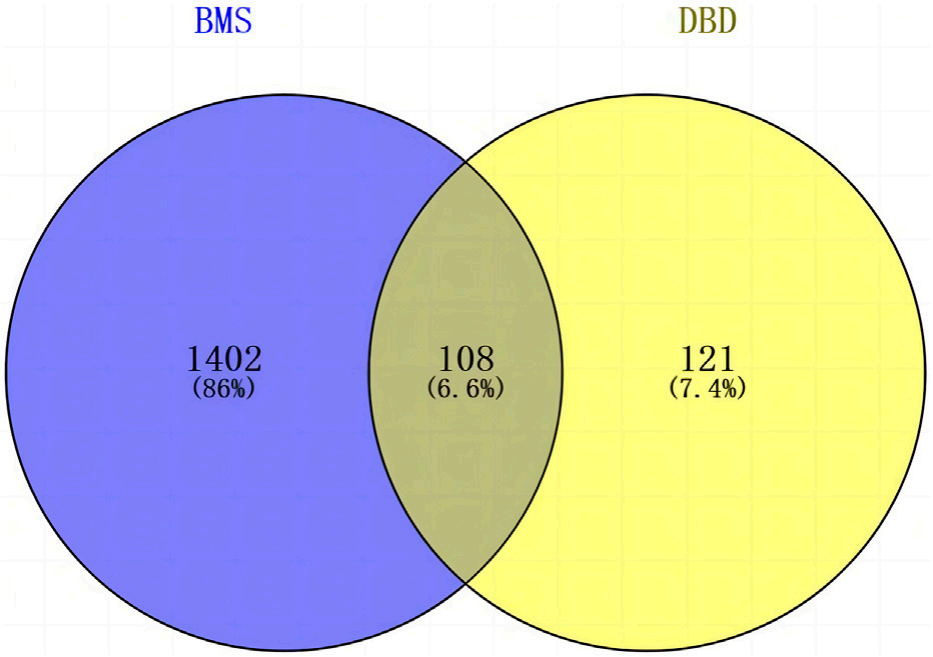
Venny diagram of BMS targets and DBD targets.

**FIGURE 4:**
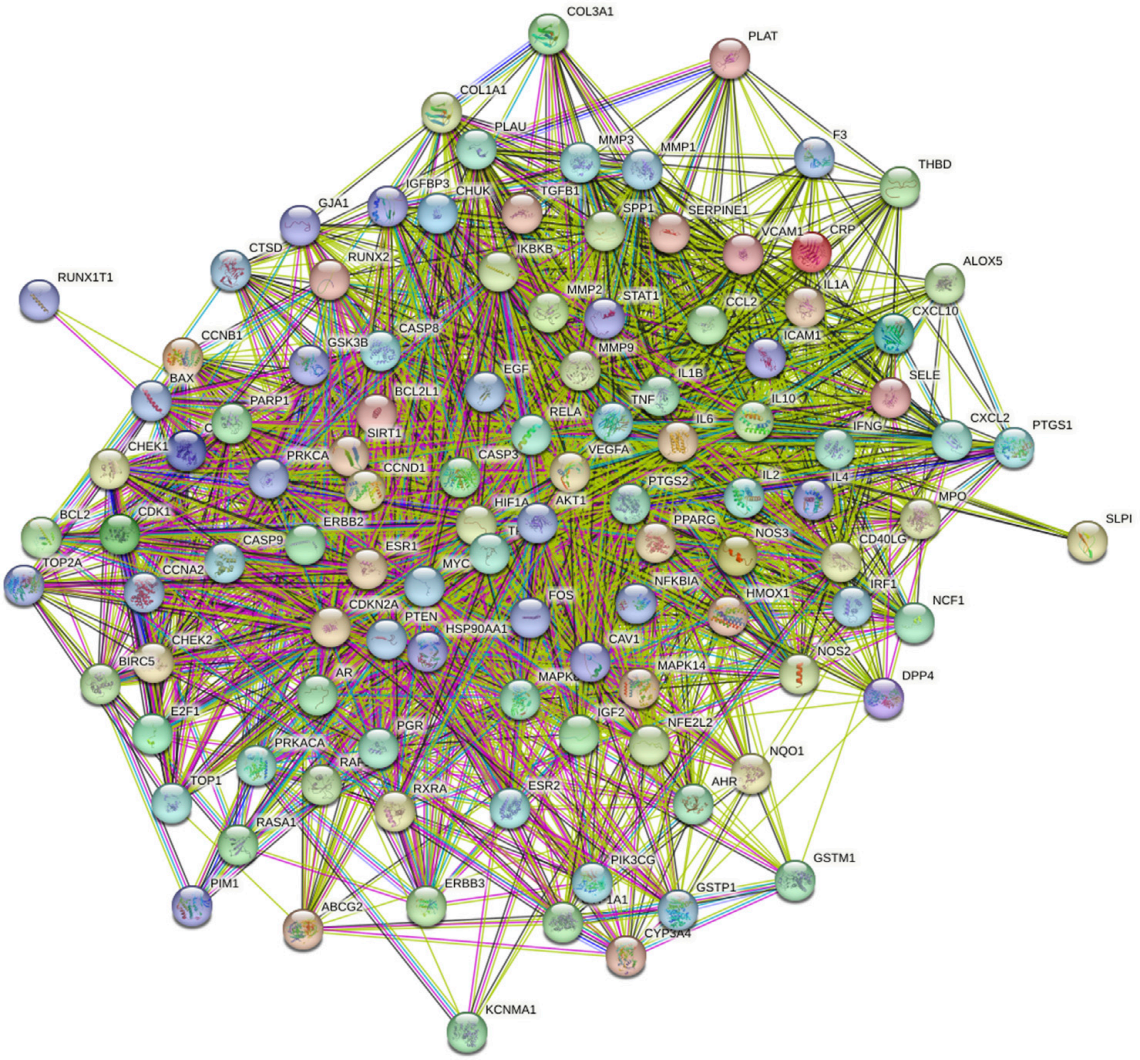
PPI network common targets of DBD and BMS. Note: Each node represents a particular protein. The edges of each node showed the innate relationships between the proteins, and the hues from yellow to red signified tiny to big values. A line between proteins served as a visual cue that they were related. The link between the proteins is closer the more lines there are between them.

**FIGURE 5:**
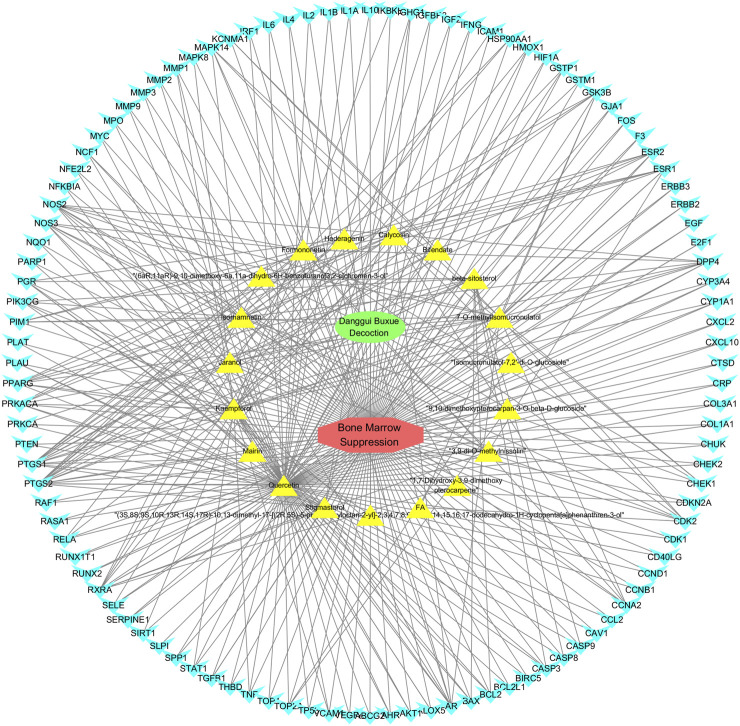
Drug-ingredient-target-disease network diagram for DBD against BMS. Note: In the network diagram, green represents Danggui Buxue Decoction (DBD), red represents Bone Marrow Suppression (BMS), yellow represents the active ingredients of DBD, and blue represents their common genes. The more lines, the closer the connection between them.

The screened 229 DBD targets and 1510 BMS targets were entered into the Venny online platform, and 108 intersection targets were obtained, accounting for 6.6%. The intersection targets were used as potential targets for DBD treatment of diseases (Figure 3). The potential targets were input into the STRING platform to obtain the PPI network (Figure 4). To understand the interaction between the targets more clearly and see the core targets more intuitively according to the link degree value, the potential intersection targets and other data were imported into Cytascape software to construct the active component-disease-target network (Figure 5).

#### 3.2.3 GO function and KEGG pathway enrichment analysis

**FIGURE 6:**
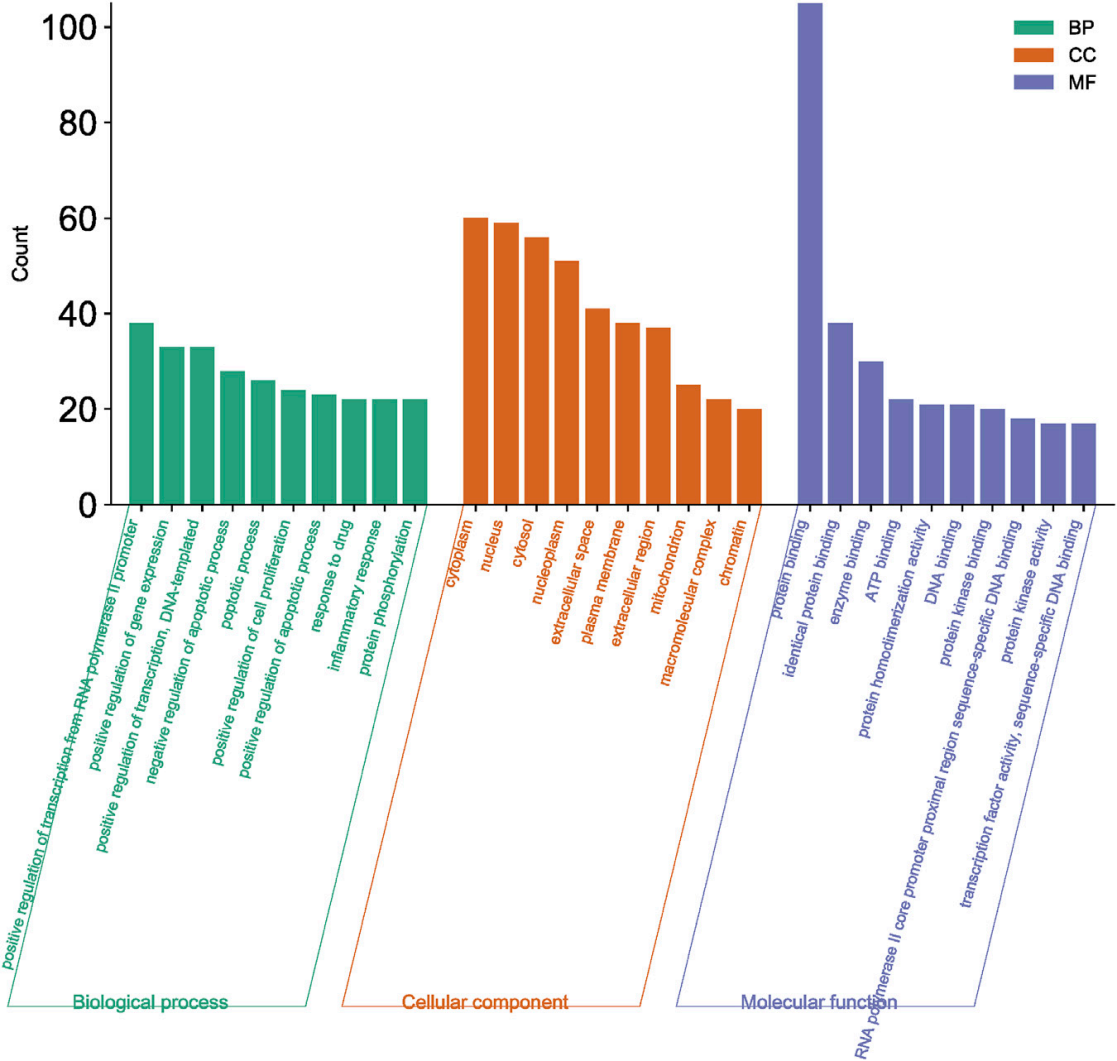
GO functional enrichment analysis of key target protein genes.

**FIGURE 7:**
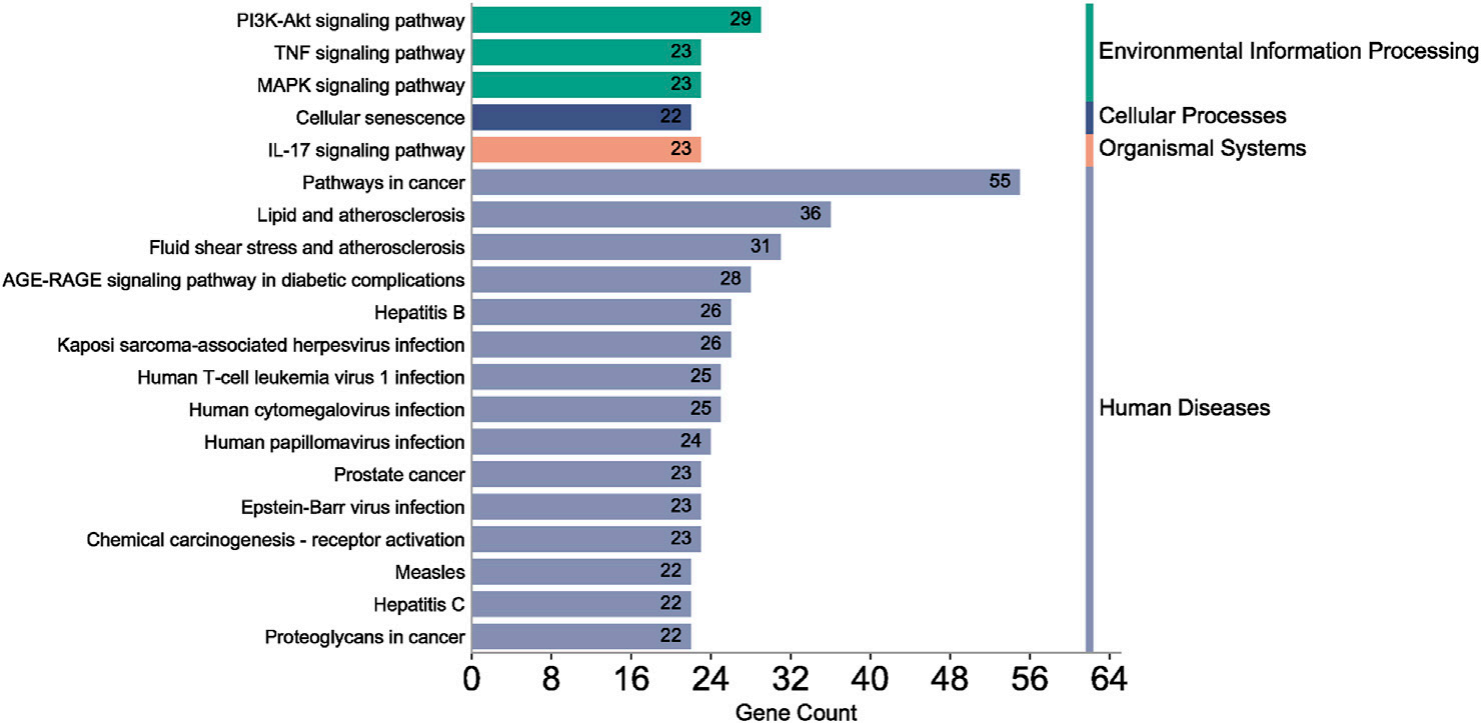
KEGG pathway enrichment analysis of key target protein genes.

A total of 108 key target protein genes were imported into the DAVID database, and data with *p* < 0.05 were selected for statistical significance. A total of 570 biological processes, 49 cellular components, 96 molecular functions, and 151 KEGG signaling pathways were involved. The top 20 items based on count were imported into the Bioinformatics platform to draw GO enrichment analysis and KEGG pathway maps. The results can be seen in Figures 6, 7. The GO enrichment analysis map showed that the essential biological process (BP), cellular component (CC) and molecular function (MF) involved in DBD acting on diseases include the positive regulation of RNA polymer II promoter transcription and the positive regulation of gene expression, cytoplasm and nucleus, protein binding and idential protein binding, respectively. From the KEGG pathway enrichment analysis map, the key KEGG pathways were enriched in cancer pathway, AGE-RAGE signaling pathway in diabetic complications, PI3K-AKT signaling pathway, TNF signaling pathway, MAPK signaling pathway, IL-17 signaling pathway, and so on.

#### 3.2.4 Molecular docking

According to the active ingredients-disease-target network, the top five active ingredients and key targets with a degree value are Quercetin, Kaempferol, Isorhamnetin, Formononetin, 7-O-methylisochronotropol, and PTGS2, PTGS1, RXRA, NOS2, as well as PRKACA. The molecular docking results are shown in Table 7, where the binding energy of Kaempferol and PTGS2 is the largest, at −10.08 kJ · mol^−1^. At the same time, the absolute values of binding energy between Quercetin and PTGS2, NOS2, and PRKACA are all above 9.0 kJ mol^−1^, with better binding ability. In addition, we found that the absolute values of all the free binding energies obtained were far more significant than 5 kJ mol^−1^, indicating that the compound had a strong binding activity with the target.

**TABLE 7:** The results of molecular docking.

Target	PDB ID	Target structure	Active ingredients	Affiffiffinity (kJ·mol^-1^)	Best-docked complex (2D)
PTGS2	5IKQ	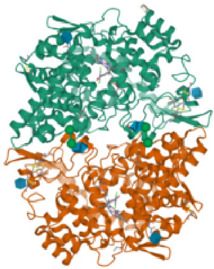	Quercetin	−9.86	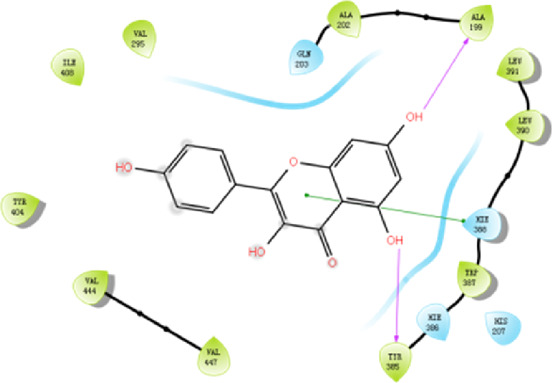
			Kaempferol	−10.08	
			Isorhamnetin	−8.91	
			Formononetin	−7.90	
			7-O-methylisomucronulatol	−7.04	
PTGS1	6Y3C	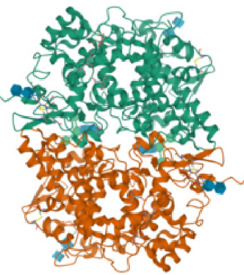	Quercetin	−8.92	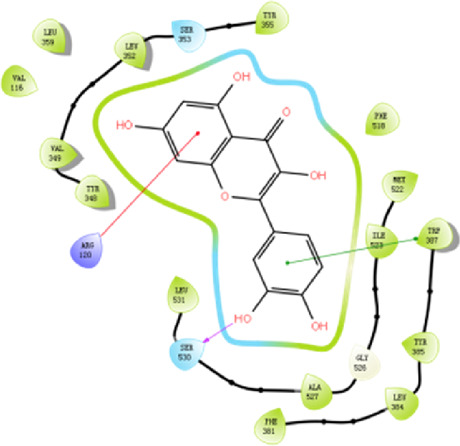
			Kaempferol	−8.23	
			Isorhamnetin	−8.60	
			Formononetin	−8.48	
			7-O-methylisomucronulatol	−8.04	
RXRA	3FAL	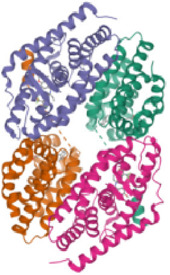	Quercetin	−8.89	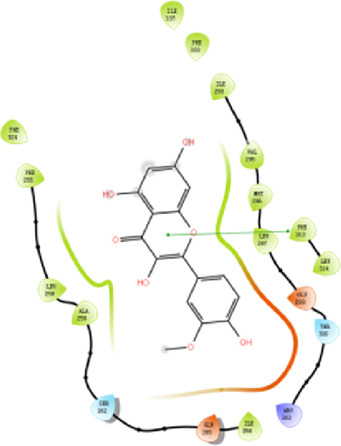
			Kaempferol	−8.21	
			Isorhamnetin	−8.90	
			Formononetin	−8.11	
			7-O-methylisomucronulatol	−7.84	
NOS2	3E7G	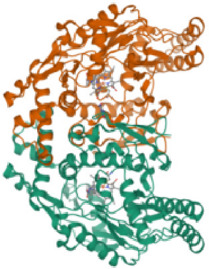	Quercetin	−9.73	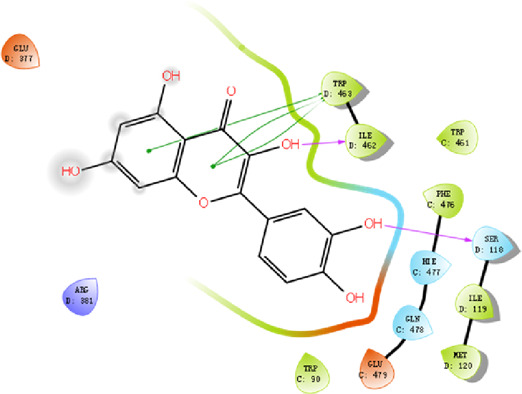
			Kaempferol	−8.98	
			Isorhamnetin	−9.47	
			Formononetin	−8.70	
			7-O-methylisomucronulatol	−8.92	
PRKACA	2UW3	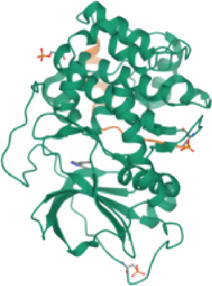	Quercetin	−9.46	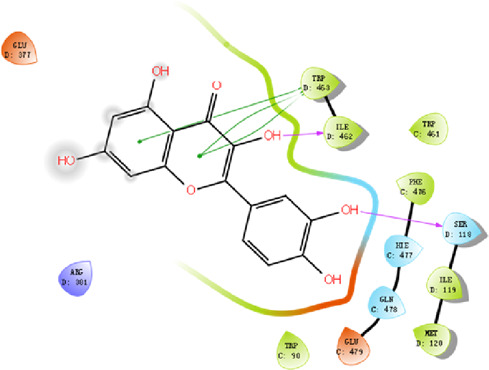
			Kaempferol	−9.01	
			Isorhamnetin	−8.52	
			Formononetin	−8.00	
			7-O-methylisomucronulatol	−8.38	
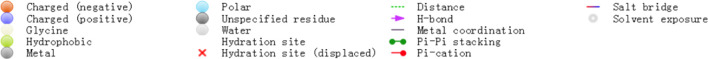					

#### 3.2.5 ADMET profiling results

The further development and use of core ingredients in Chinese medicine also require an assessment of the absorption, distribution, metabolism, and excretion (ADME) of different monomer components. The SWISS ADME database and the admetSAR database were used to analyze the ADME analysis of core ingredients, and the results showed that all ingredients had satisfactory pharmacokinetic properties (Table 8). The ADMET analysis results showed that some core active ingredients had slight toxic or no side effects in their pharmacokinetic properties. Quercetin and 7-O-methylisomucronulatol had the least toxic side effects, and there was no liver or kidney toxicity, while kaempferol and formononetin had more toxic side effects. The ADME characteristics of core active ingredients in disparate patterns, such as BBB permeant, P-gp substrate, CYP1A2 inhibitor, CYP2D6 inhibitor, and CYP3A4 inhibitor, showed positive results in some potential ingredients, indicating their ability to serve as candidate drugs, such as quercetin, isorhamnetin, and 7-o-methylisocycloaldol.

**TABLE 8:** ADMET profiling results of core ingredients.

Ingredients	Quercetin	Kaempferol	Isorhamnetin	Formononetin	7-O-methylisomucronulatol
GI absorption	High	High	High	High	High
BBB permeant	No	No	No	Yes	Yes
P-gp substrate	Yes	No	No	No	No
CYP1A2 inhibitor	Yes	Yes	Yes	Yes	Yes
CYP2C19 inhibitor	No	No	No	No	No
CYP2C9 inhibitor	No	No	No	No	No
CYP2D6 inhibitor	Yes	Yes	Yes	Yes	Yes
CYP3A4 inhibitor	No	Yes	Yes	Yes	Yes
Toxicity					
Acute oral toxicity	III	II	III	III	III
Ames mutagenesis	In-active	Active	In-active	In-active	In-active
Carcinogenicity	In-active	In-active	In-active	In-active	In-active
Hepatotoxicity	In-active	Active	In-active	Active	In-active
Nephrotoxicity	In-active	In-active	In-active	Active	In-active

## 4 Discussion

### 4.1 Analysis of adverse reactions/events of chemotherapy drugs

#### 4.1.1 Screening of adverse reactions/events

The CHPS system and the National Adverse Drug Reaction Monitoring System were used to identify 209 tumor patients who had adverse reactions/events to chemotherapy medicines for this investigation. The hospital recorded fewer adverse reactions/events from 2015 to 2017, however after 2017, they rose. This is primarily because most medical institutions lacked information service systems that could interface with the national monitoring system and all adverse reactions/events reported prior to 2018 were spontaneously conducted by medical institutions and required the use of the National Adverse Drug Reaction Monitoring information system. More importantly, there were problems with non-standard and missing reports, and the entire reporting and filing procedure was complicated, time-consuming, and arduous (Zhao et al., 2021). The previous State Food and Drug Administration of China, consequently, started a pilot project to build adverse drug reaction monitoring outposts in 2016 to address the aforementioned issues. In 2017, the hospital also became a sentinel facility for the monitoring of adverse medication reactions. The number of reported adverse reactions/events increased significantly after the CHPS system was formally established in 2018, and by 2022, there had been approximately 100 occurrences.

#### 4.1.2 Basic information and proportion of tumor patients

Of the 209 cancer patients included, 12.44% had non-small cell lung cancer, and 11.96% had colon cancer of the patients having adverse reactions/events. Additionally, 10.53% of patients had rectal cancer; 9.09% and 7.18% of patients, respectively, had non-Hodgkin’s lymphoma and acute non-lymphocytic leukemia. Furthermore, more than 5% of adverse reactions/events have involved nasopharyngeal carcinoma, cervical malignancy, and acute lymphocytic leukemia. As a result, during the course of clinical treatment, medical professionals should be aware of the possibility of adverse reactions/events, including new ones, brought on by the administration of chemotherapy medications to patients with this particular type of tumor disease.

#### 4.1.3 Combined medication and analysis of types of chemotherapy drugs

In this study, 151 cancer patients had the highest proportion of adverse reactions/events due to single drug use. However, there were still some patients who experienced negative effects or incidents as a result of taking several medications, including 50 patients who took two doses, seven patients who took three doses, and one patient who took four or more doses. Single-use medications increase adverse reactions/events, and chemotherapy drug efficacy will decline when tumor resistance to the therapies rises (Long et al., 2022). Combination chemotherapy, which has been increasingly employed in clinical treatment to accomplish the impact of improving efficacy and reducing toxicity (Pusuluri et al., 2019; Long et al., 2022), is a potential remedy for single chemotherapy resistance. Therefore, in clinical practice, it is crucial to thoroughly understand the indications, take into account the drug interactions and contraindications while using combination chemotherapy, and prevent and treat adverse reactions/events expertly.

The number of cases of chemotherapy drugs used in 209 tumor patients included in this study is 271. Figure 2 was showed that platinum complexes, anti-tumor metabolic drugs, and botanical anti-tumor drug chemotherapy drugs cause the most adverse reactions/events in cancer patients. Anti-tumor drugs are the most likely to cause serious adverse reactions/events in patients, similar to the results of the 2022 National Adverse Drug Reaction Monitoring Annual Report of China (National Medical Products Administration, 2023). The report also pointed out that the proportion of adverse reactions/events caused by cancer drugs is the largest, exceeding 35%. However, it did not provide a detailed description of the specific categories of cancer drugs, only suggesting that clinical needs to strengthen anti-tumor drug risk management. Oxaliplatin, cisplatin, and paclitaxel, three chemotherapeutic medicines, had the largest number and proportion of adverse reactions/events following use, according to Table 2. Platinum complexes such as oxaliplatin, cisplatin, carboplatin, etc. are currently 70%–80% likely to be utilized in clinical chemotherapy or combined chemotherapy for tumor illnesses (Xue et al., 2021; Feng et al., 2023). Moreover, there are many adverse reactions/events caused by chemotherapy drugs for many reasons, including gender, age, underlying disease, dosage, narrow drug treatment window, etc. (Xue et al., 2021). Therefore, in the clinical process of anti-tumor chemotherapy, it is necessary to strictly control and take measures to deal with possible adverse reactions/events to ensure the drug use of tumor patients.

#### 4.1.4 Analysis of clinical manifestations of adverse reactions/events in cancer patients

This study classified the cumulative system organs of adverse reactions/events by CTCAE. It can be seen that “medical examination” accounts for the largest proportion in Table 3. After reviewing electronic cases, it was found that their main clinical manifestations include a decrease in white blood cells and granulocytes and an increase in transaminases, which have caused damage to the body’s blood system and liver function (Ou et al., 2017; Cacabelos et al., 2021). Additionally, the main system organs accumulated by these adverse reactions/events also involve “respiratory, thoracic, and mediastinal diseases,” “skin and subcutaneous tissue diseases,” “gastrointestinal diseases,” etc. However, this result differs from China’s 2022 National Adverse Drug Reaction Monitoring Annual Report. The 2022 monitoring report includes all adverse reactions/events caused by drugs, so there is a difference (National Medical Products Administration, 2023). Moreover, all chemotherapy drugs are highly likely to cause new adverse reactions/events in patients. Therefore, it is equally important to strengthen the rational use of chemotherapy drugs and optimize nursing measures.

#### 4.1.5 Analysis of chemotherapy drugs for serious adverse reactions/events

This study did a statistical analysis of the top 10 chemotherapy medications that resulted in major adverse reactions/events in order to better understand the relationship between these drugs and serious adverse reactions/events. The most severe adverse reactions/events are caused by oxaliplatin and cisplatin, as shown in Tables 2, 5, and their clinical manifestations primarily include decreased neutrophil and leukocyte counts, allergies, and renal impairment. This result is similar to the study by Zhang et al. (2022), where platinum complexes mainly cause significant damage to the blood system, liver, and kidney functions, and is also basically consistent with the prevalent adverse events mentioned in the drug instructions and the results reported in the literature. Meanwhile, paclitaxel can produce a variety of adverse responses or events, the most severe of which are allergic reactions. Allergic reactions to paclitaxel have been known to be fatal, and its harm to the blood system cannot be discounted (Saggam et al., 2022). Apart from that, the primary emphasis of capecitabine and etoposide is on the circulatory system. Consequently, the blood system-particularly granulocytes, white blood cells, and platelets-is affected by the majority of the top 10 chemotherapy medications that induce adverse reactions or occurrences in patients.

After reviewing the CHPS and electronic case system, researchers found that while patients with “general” adverse reactions/events brought on by chemotherapy drugs can get better after drug withdrawal or medical treatment, those with “serious” adverse reactions/events can take much longer to get better, sometimes even after discharge. As a result, “serious” adverse reactions and incidents should receive more attention, and if necessary, combined department meetings should be held.

### 4.2 Network pharmacology

#### 4.2.1 Brief overview of DBD

The “NeiWaiShangBianHuo Lun” of Li Dongyuan is the source of DBD, which has a 5:1 ratio of Huang Qi and Danggui (Sun et al., 2022). While the former can energize Qi and fortify the exterior, the latter concentrates on activating and replenishing blood. DBD’s main effects include blood production, hematopoiesis promotion, accelerated blood cell maturation, and immune function regulation (Kwan et al., 2019; Tie et al., 2022). Numerous studies have demonstrated that DBD has protective effects on white blood cells and platelets as well as a potential protective effect on chemotherapy-induced BMS, increasing the sensitivity of chemotherapy medications and lowering chemotherapy-induced BMS (Li et al., 2017; Liu et al., 2021; Kong et al., 2022). Several studies on DBD have been conducted both domestically and internationally, but none of them have focused on the molecular mechanisms by which DBD prevents BMS.

#### 4.2.2 The active ingredients of DBD against BMS

In this study, GeneGards and OMIM databases were used to screen disease targets, and the TCMSP platform was used to screen the effective components and targets of DBD and to determine the common targets of the two. The PPI network and the active ingredient-disease-target network were subsequently built, and important targets’ GO and KEGG enrichment analyses were performed using the DAVID database. The effective ingredients of DBD ranked in the top five with the Degree value were quercetin, kaempferol, isorhamnetin, formononetin, and 7-O-methylisochronotropol. According to molecular docking results, these five active ingredients can effectively bind to these five main target proteins, with quercetin, kaempferol, and isorhamnetin having the best binding energy with the target protein. According to recent research, kaempferol-rich non-alcoholic polyphenol concentrate made from Cabernet Sauvignon grapes in Kazakhstan has a healing impact on the hematological system in acute radiation injury (Shulgau et al., 2014). However, the role of kaempferol in the concentration has yet to be determined due to the concentration’s complexity. Additionally, the primary ingredient in the Bushen Jianpi Quyu Formula, kaempferol, can prevent BMS from being alleviated by inhibiting the expression of the PI3K/AKT/NF-B signal pathway (Li et al., 2022). Through mechanisms that control HGF and HIF, quercetin can reduce the BMS that cisplatin causes, likewise, it can lessen DNA oxidative damage brought on by etoposide in rat bone marrow cells and is crucial for bone marrow cell protection (Papiez, 2014; Chuang et al., 2022). Furthermore, the findings in Table 8 also show that quercetin has minimal toxicity, no carcinogenicity, and no mutagenicity, making it a promising active ingredient for future medication development. Isorhamnetin’s function in BMS and hematopoiesis has not been the subject of any studies.

#### 4.2.3 The key targets and major signaling pathways of DBD against BMS

According to the degree ranking in the Drug-ingredient-target-disease network, the key targets are PTGS2, PTGS1, RXRA, NOS2, and PRKACA. PTGS2, also known as COX2, has been found to possess a sublethal or lethal effect on meloxicam, a selective COX2 inhibitor *γ*-irradiated mice have significant hematopoietic stimulation and survival-enhancing effects (Hofer et al., 2012). These effects are connected to meloxicam’s capacity to raise granulocyte colony-stimulating factor levels in the blood, and it is assumed that blocking the COX2 protein may be a major strategy for preventing BMS. We find that PTGS2 is a key protein in chemotherapy-induced BMS, which is also consistent with the research results of Wang et al. (2020). Besides, the molecular docking studies show that PTGS2 can have the best docking effects with quercetin and kaempferol, and its absolute value of binding energy is about 10 kJ mol^−1^. In light of these findings, PTGS2 is a potential primary target for the therapy of BMS. We also discovered that ionizing radiation does not require NOS2 to induce BMS and that RXRA ligands are present and dynamically increase in mouse hematopoietic cells (Niu et al., 2017; Li et al., 2018). According to the KEGG pathways analysis, DBD can also reduce BMS by activating the PI3K-AKT, TNF, MAPK, and IL-17 signaling pathways. By controlling the expression of the PI3K-AKT signaling pathway, a variety of pharmacological medicines and components can reduce BMS, enhance hematopoiesis and platelet production, and even play an immunomodulatory effect (Liu et al., 2010; Wang et al., 2011; Chen et al., 2023). In order to shield isolated bone marrow cells from 5-fluorouracil-induced cell death and inflammatory responses, the physiologically active glycoside Martynoside can inhibit the TNF signaling pathway (Hong et al., 2021). At the same time, the TNF signaling system may shield bone marrow cells from the harm caused by carbon ion radiation (Liu et al., 2019). Recent research has found that Specnuezhenide can enhance the hematological and immunological functions of BMS mice, and it is speculated that it can significantly help the hematopoietic and immune functions of tumor patients after chemotherapy and plays a role by increasing the expression levels of key proteins MEK and p-ERK in the MAPK signaling pathway (Han et al., 2023). Leukopenia, thrombocytopenia, erythrocytopenia, and bone marrow cell depletion are caused by radiation therapy associated with hemorrhage, which increases the production of IL-1, IL-6, and IL-17A in the IL-17 signaling pathway and increases BMS (Kiang et al., 2015).

## 5 Conclusion

The adverse effects/events that chemotherapy medications can have on patients are unavoidable and sometimes ignored in anti-tumor therapy and chemotherapy harm surely lengthens the time it takes for patients to recover. Age, tumor kind, drug type, combination therapy, and other characteristics of tumor patients all have an impact on the likelihood of chemotherapy damage. Among them, we need to focus on the damage of platinum drugs, paclitaxel, and other chemotherapy drugs to the body. At the same time, most chemotherapy drugs will cause BMS in patients. Blood routine function tests should be done on schedule when taking medication, which can improve the prognosis of patients through early detection and treatment. Further, this study has proven that DBD has the effect of preventing BMS after chemotherapy. Through network pharmacology, the effective ingredients of DBD have been analyzed, and their treatment mechanisms have been systematically studied, providing new ideas for the treatment of chemotherapy-induced BMS. The results showed that the key ingredients that increase hematopoiesis and lessen BMS damage through the control of signal pathways such as PI3K-AKT, TNF, MAPK, and IL-17 include quercetin, kaempferol, and isorhamnetin. This research will also serve as a theoretical underpinning and scientific foundation for our subsequent investigations into the effectiveness and molecular basis of self-made drug delivery microspheres for the prevention of chemotherapy-induced BMS.

## Data Availability

The datasets presented in this study can be found in online repositories. The names of the repository/repositories and accession number(s) can be found in the article/Supplementary material.
